# Dr. W. H. Wollaston

**Published:** 1829-02

**Authors:** 


### OBITUARY.

We have to announce, with the deepest regret, the death of Dr. W. H. Wollaston, which took place on the 22d December. As a man of general scientific acquirements, he held the very highest rank. His great ability was admitted not only by the learned of this country, but throughout Europe his name was always mentioned with admiration, and his opinions with confidence His works are too well known, and too justly appreciated, to demand our formal praise.

